# Bilateral Anterior Shoulder Dislocations: A Narrative Review and Case Report

**DOI:** 10.7759/cureus.95186

**Published:** 2025-10-22

**Authors:** Jack Tierney, Dominic Waugh, Amit Putti

**Affiliations:** 1 Trauma and Orthopaedics, NHS Forth Valley, Larbert, GBR; 2 Trauma and Orthopaedics, Greater Glasgow and Clyde, Glasgow, GBR

**Keywords:** orthopaedics, shoulder, sport, surgery, trauma

## Abstract

Simultaneous bilateral anterior shoulder dislocations are extremely rare and typically result from high-energy trauma or seizure activity. Due to their unusual presentation, diagnosis and associated injuries may be missed without thorough assessment and advanced imaging. Here, we present a case report with an associated narrative review of the relevant medical literature. In addition, we propose a treatment algorithm to optimise management of this cohort. A 44-year-old male presented to the emergency department with bilateral shoulder pain and loss of function after lifting a 100 kg weight overhead during a strongman competition. Initial radiographs confirmed bilateral anterior shoulder dislocations without associated fracture. Bilateral closed reductions were successfully performed under sedation. On follow-up, the patient reported numbness in the posterior left forearm. Bilateral shoulder MRI revealed full-thickness supraspinatus and infraspinatus tears, with a partial subscapularis tear and biceps tendon subluxation on the left. The patient was listed for staged arthroscopic rotator cuff repair, beginning with the dominant (left) shoulder. A narrative review of 33 recent case reports was conducted. Seizure was the most common cause of bilateral dislocation, and anterior dislocations predominated. Concomitant injuries, particularly fractures and soft tissue damage, were common. However, MRI was not often used, possibly contributing to an under-reporting of rotator cuff pathology. Functional outcomes were generally good with appropriate management, although persistent deficits were more common in patients with associated injuries. This case reinforces the importance of comprehensive clinical and radiological assessment in bilateral shoulder dislocations. Our review evaluates current imaging and management and highlights that concomitant fractures and soft tissue injury, including rotator cuff tears, are common, but may be under-recognised without advanced imaging. Our treatment algorithm advocates for a high index of suspicion and emphasises the importance of considering advanced imaging techniques in both the acute and outpatient settings.

## Introduction

Shoulder dislocation (SD) is a common presentation in emergency departments, accounting for approximately 50% of all major traumatic joint dislocations [1], with an incidence of 8-17 cases per 100,000 population per year [2]. Of these, unilateral anterior SD is the most common, representing up to 95% of cases [3,4]. Recurrence rates following a true dislocation vary widely, with reported rates ranging from 85% to 92% [5]. In contrast, bilateral SDs (BSDs) are rare, with an estimated incidence of 0.6 per 100,000 population [6,7]. Unlike unilateral dislocations, which are typically anterior, BSDs tend to occur posteriorly and are often associated with seizures or electrical trauma [3]. BSDs can be broadly classified as those occurring in the same direction (bilateral symmetric SD or BSSD) or in different directions (bilateral asymmetric SDs or BASD), depending on the direction of each SD. There are three recognised subtypes of BSSD: posterior, anterior, and inferior [7]. BASD is classified as either anteroinferior (one side anterior with the other side inferior) or anteroposterior (one side anterior with the other side posterior) [7]. We present the case of a 44-year-old male who sustained simultaneous bilateral anterior SDs with associated full-thickness bilateral rotator cuff tears during participation in a strongman competition.

## Case presentation

**Figure 1:**
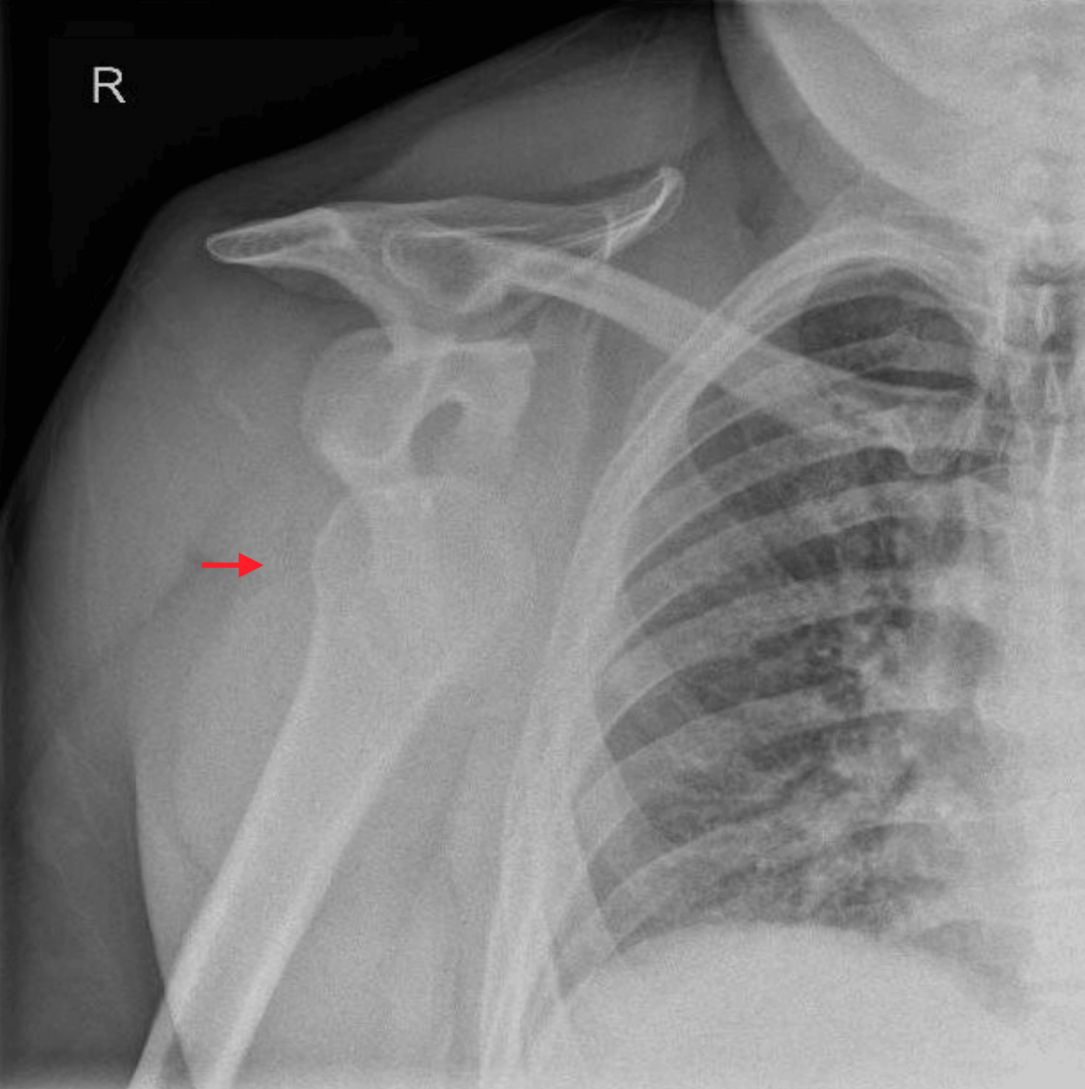
Right shoulder radiograph Anteroposterior radiographs of the right shoulder demonstrating anterior glenohumeral dislocation with inferomedial displacement of the humeral head relative to the glenoid (red arrow). No associated fracture is identified. Surgical anchors from previous arthroscopic rotator cuff repair are visible overlying the humeral head.

**Figure 2:**
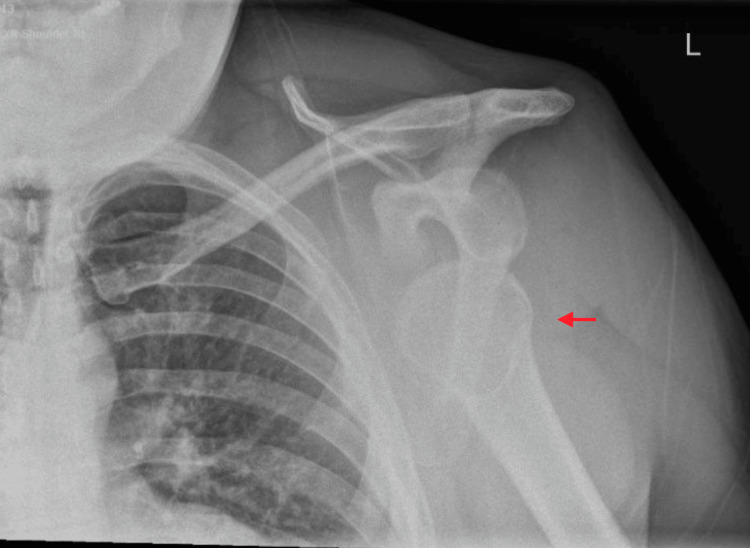
Left shoulder radiograph Anteroposterior radiograph of the left shoulder demonstrating anterior glenohumeral dislocation with inferomedial displacement of the humeral head relative to the glenoid (red arrow). No associated fracture is identified.

A 44-year-old male presented to the emergency department with bilateral shoulder pain and inability to move either arm following participation in a strongman competition. He reported losing control while lifting a 100 kg weight overhead, which subsequently fell behind him, resulting in forced hyperextension of both shoulders. This was associated with an audible 'popping' sensation, visible deformity of both shoulder contours, and immediate functional loss. His past medical history included a previous right arthroscopic rotator cuff repair for an infraspinatus tear. He was otherwise fit and well, with no regular medications. Social history noted occasional self-reported recreational marijuana use. En route to the hospital, he received nitrous oxide analgesia administered by paramedics. On arrival, the patient was in significant pain and positioned prone for comfort. An initial assessment was performed by the emergency medicine team, and bilateral shoulder radiographs confirmed symmetrical anterior bilateral SDs (Figures 1, 2)*.*

**Figure 3:**
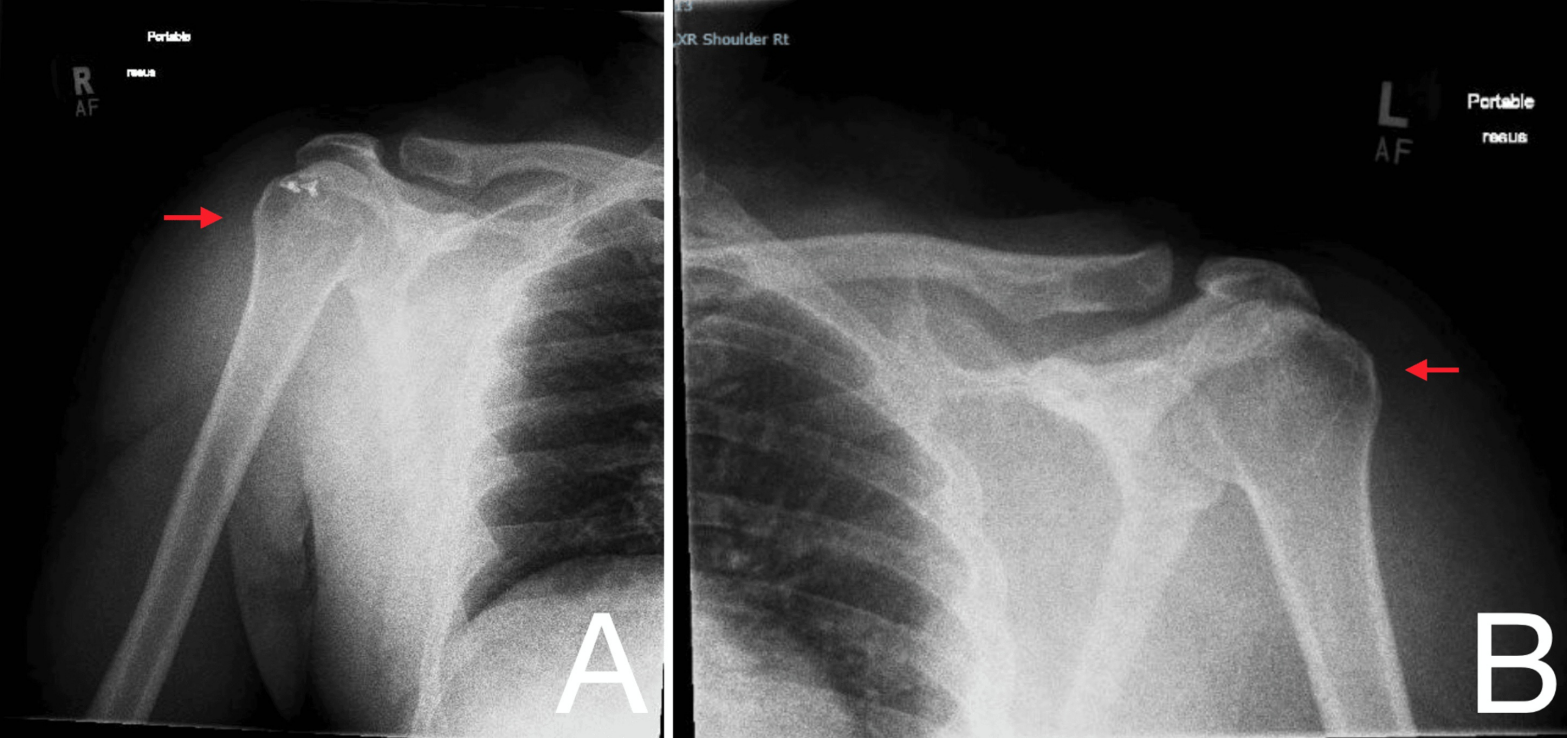
Bilateral shoulder radiographs Post-reduction anteroposterior radiographs of the right (A) and left (B) shoulders demonstrating successful reduction of bilateral anterior glenohumeral dislocations (red arrows).

Due to the severity of pain, procedural sedation was administered using fentanyl, propofol, and ketamine. Closed reduction was successfully performed bilaterally by the orthopaedic registrar using traction-countertraction techniques. Post-reduction examination under sedation demonstrated no instability in either shoulder, with a full passive range of motion. Following recovery from sedation, neurovascular examination revealed intact motor and sensory function in the axillary, median, ulnar, and radial nerve distributions bilaterally. Post-reduction radiographs confirmed successful relocation of both glenohumeral joints (Figure 3). The patient was discharged with bilateral slings, appropriate analgesia, and a plan for outpatient orthopaedic review within two to three weeks.

**Figure 4:**
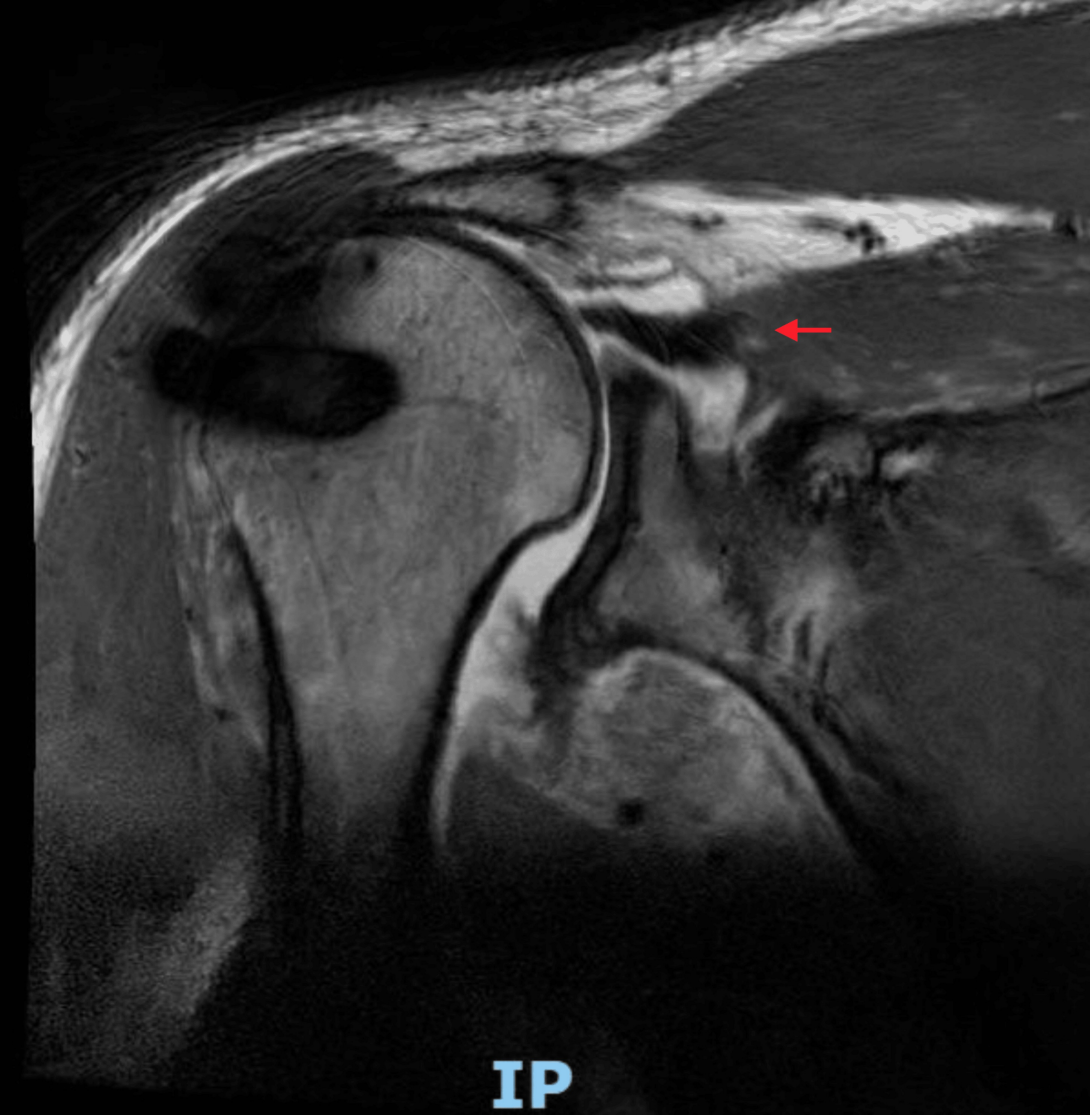
MRI right shoulder (coronal slice) A full-thickness tear of the supraspinatus tendon is apparent (red arrow). Post-surgical changes from previous rotator cuff repair are evident, with associated artefact. There is mild humeral head subluxation contributing to subacromial impingement.

**Figure 5:**
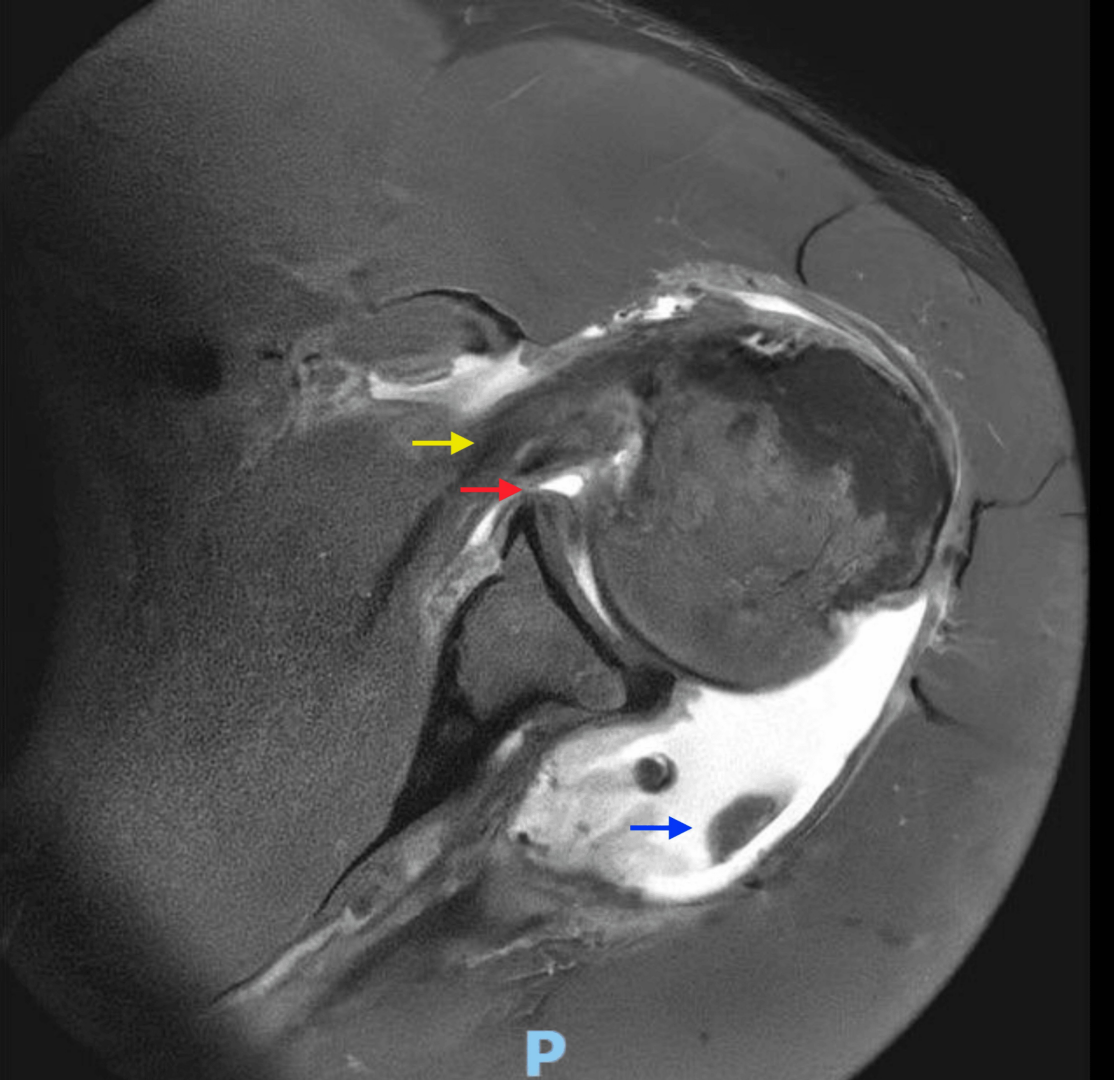
MRI left shoulder (axial slice) Findings demonstrate complete full-thickness tears of the infraspinatus muscle (blue arrow). There is also significant subscapularis tendinitis with a partial tear (yellow arrow), in addition to an extensive anterior labral tear (red arrow).

The patient was subsequently reviewed in the outpatient orthopaedic clinic. Clinical examination revealed intact sensation in the axillary, radial, median, and ulnar nerve distributions of the right arm. However, he reported numbness over the posterior aspect of the left forearm. Given the high-energy mechanism of injury, urgent bilateral shoulder magnetic resonance imaging (MRI) was arranged to evaluate for soft tissue injury, particularly rotator cuff pathology. MRI of the right shoulder confirmed full-thickness tears of the supraspinatus and infraspinatus tendons, with preservation of the subscapularis tendon (Figure 4). The left shoulder MRI demonstrated full-thickness tears of the supraspinatus and infraspinatus, a partial subscapularis tear, and subluxation of the long head of the biceps tendon (Figure 5).

The patient was recalled to the clinic for discussion of results and further management planning. At outpatient review, the patient demonstrated good active range of motion in both shoulders, with flexion to approximately 120°, extension to 90°, and abduction to 90° bilaterally. External rotation was notably restricted in both shoulders, whereas internal rotation was preserved. Neurovascular examination remained intact. Given the patient’s age and functional requirements, bilateral arthroscopic rotator cuff repair was considered. However, the delayed presentation (seven weeks post-injury) and evidence of a re-tear in the previously repaired right rotator cuff complicated surgical planning. Additional concerns were raised regarding anaesthetic risks due to the anticipated prolonged operative time. A staged surgical approach was therefore recommended. The left rotator cuff repair, corresponding to the patient’s dominant side, was planned as the initial procedure, followed by right rotator cuff repair four to six weeks later. The patient was counselled on the benefits of surgical repair, including tendon healing and improved function, as well as potential risks such as infection, scar tenderness, nerve or vascular injury, re-tear, anchor failure, shoulder stiffness, residual pain, long-term osteoarthritis, deep vein thrombosis, pulmonary embolism, and general medical complications. Following informed consent, the patient was listed for left arthroscopic rotator cuff repair.

## Discussion

Narrative review of the literature

Aim

The study aimed to contextualise a rare case of simultaneous BSD through a narrative review of published case reports, identifying patterns in imaging and management strategies.

Eligibility Criteria

We included case reports and case series describing simultaneous BSDs, with or without associated soft tissue or bony injuries. Only English-language articles published within the last 10 years were considered. Studies exclusively focusing on unilateral dislocations or chronic instability were excluded.

Information Sources and Search Strategy

A comprehensive literature search was performed using MEDLINE and EMBASE databases. The search strategy combined terms related to: (bilateral OR simultaneous) (dislocation* OR subluxation) (shoulder* OR glenohumeral)

Boolean operators and adjacency terms refined the search results. The full search strategy is detailed in the appendix. *Search Results and Study Selection*

**Figure 6:**
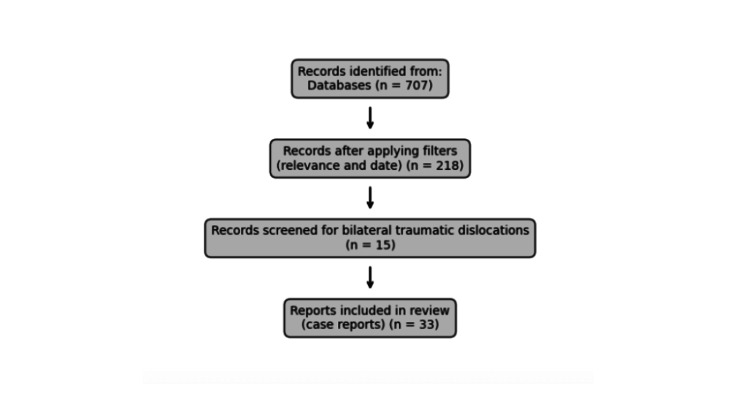
Literature search flow diagram

The initial search yielded 707 articles (Search 8). After applying filters for relevance to complications, management, and outcomes (Search 10), and limiting to the last 10 years (Search 11), 218 articles remained. Further screening focused on simultaneous BSDs (Search 12) and traumatic or acute presentations (Search 14), yielding 15 articles. Manual full-text screening expanded inclusion to 33 case reports. A complete inclusion list is provided in the appendix*.* A literature search flow diagram is shown in Figure 6.

Data Summary

Data extracted included patient demographics, injury mechanism, imaging modalities, associated injuries, management approaches, and outcomes. Findings were synthesised narratively, with particular attention to the role of advanced imaging in detecting concomitant injuries. A summary of papers is included in the appendix*.* *Risk of Bias and Limitations*

As a narrative review of case reports, the findings are inherently descriptive and prone to reporting bias. Variability in clinical detail and imaging techniques limited direct comparisons across studies.

Discussion

Demographics and Aetiology

Among the identified case reports, there was a clear male predominance, with 26 males [3,8-31] and 8 females [4,32-38]. The mean age at injury was 42 years, with males presenting at a younger mean age (38 years) compared with females (56 years). All cases involved a traumatic aetiology. Notably, seizures accounted for the largest subgroup (n = 20) [8,11-13,15-18,21,22,24,25-27,29-31,34,36,37]. This was followed by sport-related injuries (n = 5) [3,14,23,28,33], falls (n = 3) [4,32,38], road traffic collisions (n = 2) [20,35], accidental electrocution (n = 1) [19], defibrillation (n = 1) [9], and physical assault (n = 1) [10]. Aetiological distribution is summarised in Table 1.

**Table 1:** Aetiology of bilateral shoulder dislocation RTC: Road traffic collision. Data adapted from [3, 4, 8-38].

Mechanism	Number of patients	Percent of total case reports
Seizure	20	61%
Sports	5	15%
Falls	3	9%
RTC	2	6%
Electrocution	1	3%
Defibrillation	1	3%
Assault	1	3%

The predominance of seizure-induced dislocations underscores the susceptibility of the glenohumeral joint to violent involuntary muscle contractions. A previous systematic review identified seizures as the most common cause of bilateral anterior SDs [1]. However, our case aligns with the less common sport-related injuries, particularly those involving arms extended overhead, which account for only 5.7% of bilateral SDs [1]. Anterior dislocations were the most frequent type observed (n = 25) [3,4,8-12,14-16,20,21,23-25,27-30,32-34,36-38], followed by posterior dislocations (n = 4) [13,18,19,22], and inferior dislocations (n = 2) [26,35]. Two cases of asymmetrical SDs were reported, both describing anterior dislocation of the right shoulder with posterior dislocation of the left shoulder [17,31]. These findings are summarised in Table 2.

**Table 2:** Direction of bilateral shoulder dislocation Data adapted from [3, 4, 8-38].

Direction	Number of patients	Percent of total case reports
Anterior	25	76%
Posterior	4	12%
Inferior	2	6%
Asymmetrical	2	6%

Investigation Use

Diagnostic imaging and investigation methods were specified in 32 of the 33 reports. Standard radiographs were consistently employed for initial diagnosis and post-reduction confirmation in nearly all cases (n = 32) [3,4,8-33,35-38]. Computed tomography (CT) was used in 13 cases [10,12,13,18,19,22,24,25,27,29,31,32,36], primarily to evaluate associated fractures or complex injury patterns. MRI was used in a limited number of cases (n = 3) [24,33,35] to assess for rotator cuff tears and other soft tissue injuries. Electromyography was employed in three cases [8,36,38] to investigate nerve injuries. A summary of employed investigations is provided in Table 3.

**Table 3:** Utilisation of investigations in bilateral shoulder dislocation CT: Computed tomography; EMG: Electromyography; MRI: Magnetic resonance imaging. Data adapted from [3,4,8-33,35-38].

Imaging technique	Number of patients	Percent of total case reports
X-ray	32	97%
CT	13	36%
MRI	3	9%
EMG	3	9%
Unreported	1	3%

Associated Injury

Concomitant injuries were common with bilateral SD, although a limited number of reports (n = 9) [4,8,10,14-16,20,23,27] documented dislocations without associated fracture or soft tissue injury. The most frequently reported concomitant injury was proximal humeral fracture (N = 20) [3,9,11,13,17-19,21,22,24-26,28-34,36]. Among these, the majority sustained bilateral fractures (n = 18), while the remainder had unilateral fractures (n = 2). Other injuries included Hill-Sachs lesions (n = 6) [12,13,19,22,28,37] and a coracoid fracture [12]. These findings are summarised in Table 4.

**Table 4:** Bony injuries associated with bilateral shoulder dislocation Data adapted from [3, 4, 8, 10-34, 36, 37].

Injury	Number of patients	Percent of total case reports
Bilateral proximal humerus fracture	18	55%
Unilateral proximal humerus fracture	2	6%
Hill-Sachs	6	18%
Coracoid fracture	1	3%
No associated injury	9	9%

Soft tissue injuries were reported less commonly. Two cases described rotator cuff tears: one isolated [35] and another concomitant with a proximal humeral fracture [28]. Additionally, there was a single reported rupture of the long head of the bicep tendon [12]. This is summarised in Table 5.

**Table 5:** Soft tissue injuries associated with bilateral shoulder dislocation Data adapted from [12, 28, 35].

Injury	Number of patients	Percent of total case reports
Rotator cuff tear	2	6%
Long head biceps tear	1	3%
No reported soft tissue injury	30	91%

The low reporting rate of soft tissue injuries may reflect underdiagnosis rather than true rarity, particularly given the limited use of MRI across the reviewed cases. Previous studies have demonstrated that in patients over 40 years of age, the incidence of rotator cuff tears following unilateral SD ranges from 30% to 50% [39]. In our case, bilateral full-thickness supraspinatus tears were identified on MRI, underscoring the importance of considering soft tissue injuries even in the absence of associated fractures. Neurological injuries were described in two cases, both following bilateral anterior dislocation. One case involved a unilateral axillary and radial nerve injury with complete recovery at six-month follow-up [38], while the other reported unilateral brachial plexus neurapraxia, with resolution of paraesthesia by 12 months [36]. These findings are summarised in Table 6*.*

**Table 6:** Neurological injuries associated with bilateral shoulder dislocation Data adapted from [36, 38].

Injury	Number of patients	Percent of total case reports
Axillary and radial nerve	1	3%
Brachial plexus	1	3%
No reported nerve injury	30	94%

At clinical follow-up, our patient reported numbness over the posterior aspect of the left forearm. This, like the low rates of reported soft tissue injuries, reinforces the potential for neurovascular compromise in bilateral SDs and the importance of a thorough neurological examination. All patients should undergo careful clinical and radiological assessment to exclude associated fractures, rotator cuff tears, and nerve injuries, which, if missed, may contribute to significant morbidity.

Management

Bilateral SDs were most commonly managed by closed reduction, which was attempted in 22 cases. Of these, 15 cases underwent closed reduction alone [3,4,14-16,19-21,23,25,27,35-38] without the need for operative intervention. In one case, closed reduction was successful unilaterally, with the contralateral shoulder requiring open reduction [30]. In another case, closed reduction was performed with contralateral open reduction and internal fixation (ORIF) [31]. The site of attempted closed reduction was specified in eight cases. Of these, five cases were performed in the emergency department [15,16,25,28,35], and three were taken to theatre [19,30,31]. In the remaining cases (N = 14), the location of closed reduction was not reported. Of these, eight cases were performed under sedation or analgesia [3,4,20,21,23,24,30,37], including a single case in which each shoulder was reduced at separate occasions due to missed contralateral dislocation [4]. A limited number of reductions (n = 3) were performed under general anaesthesia [14,26,36]. The anaesthetic method was not reported in three cases [27,32,33]. It is noted that in a single case [33], closed reduction had been performed at another hospital, with a delayed diagnosis of bilateral greater tuberosity fractures made at a subsequent clinic review. Open reduction was necessary in two reports: one unilateral case following failed closed reduction [30] and another involving bilateral open reduction [8]. Six cases underwent closed reduction followed by ORIF: in five reports, closed reduction preceded subsequent ORIF [24,26,28,32,33] with a single case requiring ORIF following an iatrogenic fracture sustained during closed reduction [32]. Fracture-dislocation patterns frequently required ORIF, which was performed in 10 cases [9-13,17,18,22,29,34]. These findings highlight the risks associated with forceful closed reduction attempts and underscore the importance of appropriate sedation, patient positioning, and reduction technique [40]. Collectively, the evidence supports an initial attempt at closed reduction when feasible, reserving open surgical management for irreducible dislocations or those with complex fracture patterns. Treatment approaches are summarised in Tables 7, 8*.*

**Table 7:** Treatment strategies in bilateral shoulder dislocation ORIF: Open reduction and internal fixation. Data adapted from [3,4,8-38].

Strategy	Number of patients	Percent of total case reports
Closed reduction (no ORIF)	15	46%
Open reduction (no ORIF)	2	6%
Closed reduction + ORIF	6	18%
ORIF	10	30%

**Table 8:** Site of closed reduction Data adapted from [3,4,14-16,19-21,23-28,30-33,35-38].

Site	Number of patients	Percent of total case reports
Emergency department	5	15%
Operating theatre	3	9%
Not reported	14	42%

Post-reduction Immobilisation

Post-reduction immobilisation techniques were specified in 21 reports. The most commonly used method was a simple sling, applied in 12 cases [3,8,11,12,15-17,20,22,29,33,38]. Velpeau slings were employed in four cases: bilaterally in three [13,27,32] and unilaterally in one, where it was combined with a contralateral abduction splint [28]. Orthoses or structured splints were used in three reports [14,19,31], typically positioning the arm in adduction and internal rotation. Abduction splints were applied unilaterally in one case [28] and bilaterally in another [21]. Immobilisation by strapping the hands to the chest was described in a single case [24]. Immobilisation techniques were largely individualised based on injury pattern, reduction stability, and the presence of associated fractures or soft tissue damage. A summary of these approaches is provided in Table 9.

**Table 9:** Immobilisation strategies in bilateral shoulder dislocation Data adapted from [3,8,11-17,19-22,24,27-29,31-33,38].

Immobilisation technique	Number of patients	Percent of total case reports
Sling	12	36%
Velpeau sling	4	12%
Orthoses splint	3	9%
Abduction splint	2	6%
Strapping	1	3%
Not specified	12	36%

Functional outcomes were reported in 27 cases, with outcome data unavailable in four reports. The majority (n = 24) demonstrated good bilateral functional recovery without significant restriction in range of motion (ROM) or persistent pain [3,4,8,11-13,16-22,24-29,30,33,36-38]. Notably, two reports described bilateral ROM limitations [32,35], and one case documented unilateral restriction complicated by osteomyelitis and osteolysis [31]. The duration and type of immobilisation varied across cases. However, existing evidence suggests that immobilisation for more than one week does not reduce recurrence rates of dislocation [41]. Overall, the findings indicate that, with timely diagnosis, appropriate treatment, and structured rehabilitation, most patients can expect good functional recovery. Nonetheless, residual stiffness and weakness may occur, particularly in cases involving associated fractures or soft tissue injuries.

Proposed Treatment Algorithm

**Figure 7:**
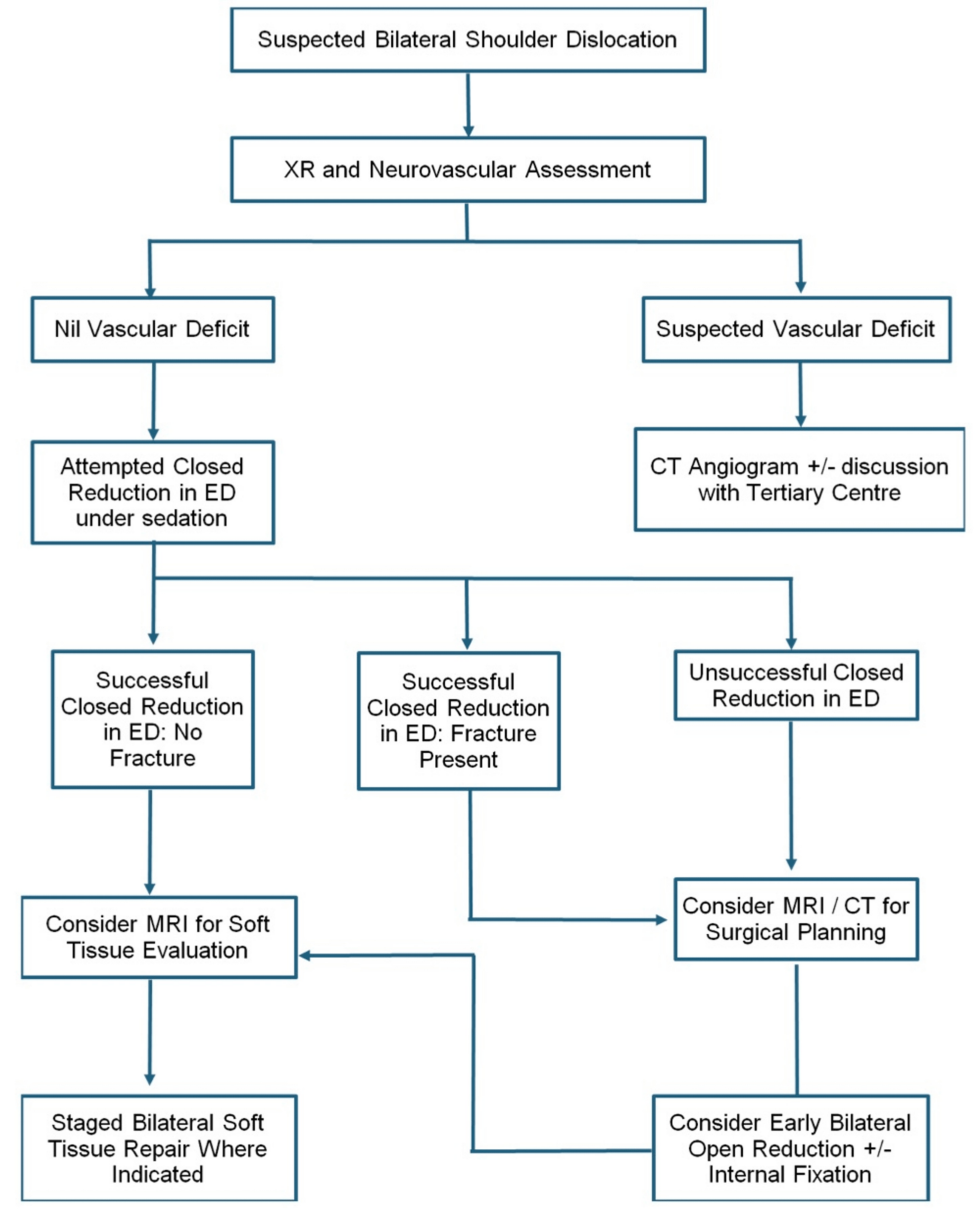
Bilateral shoulder dislocation proposed treatment algorithm CT: Computed tomography; ED: Emergency department; MRI: Magnetic resonance imaging; XR: X-ray.

Given these findings, the authors propose a treatment strategy for bilateral SD summarised in Figure 7*.* We advocate thorough neurovascular assessment at the time of assessment with initial X-ray imaging to determine associated fracture patterns. In cases where fractures raise concerns about vascular compromise, further evaluation with CT angiography should be performed, with urgent consultation from orthopaedic and vascular surgery teams as appropriate. Such cases should be managed at centres equipped to provide multidisciplinary care. Closed reduction attempts should be performed in the emergency department under appropriate sedation whenever feasible. If closed reduction is unsuccessful, CT imaging is advised before operative intervention to assist with surgical planning for ORIF or replacement. Furthermore, given the potential for under-reporting of soft tissue injuries in the literature, we advocate for the consideration of MRI imaging in the outpatient setting to assess for rotator cuff tears and other soft tissue damage. This comprehensive approach aims to optimise functional outcomes while minimising complications in this rare but complex injury pattern.

## Conclusions

Simultaneous bilateral anterior SDs are rare injuries where functional outcomes are generally favourable with prompt recognition, appropriate imaging, and shoulder-specific immobilisation and rehabilitation. However, as these injuries are commonly associated with fractures, soft tissue damage, and, in some cases, neurovascular compromise, comprehensive imaging, especially in patients with concerning symptoms, is essential to optimise outcomes in this rare but potentially complex injury pattern.

## Figures and Tables

**Figure 8:**
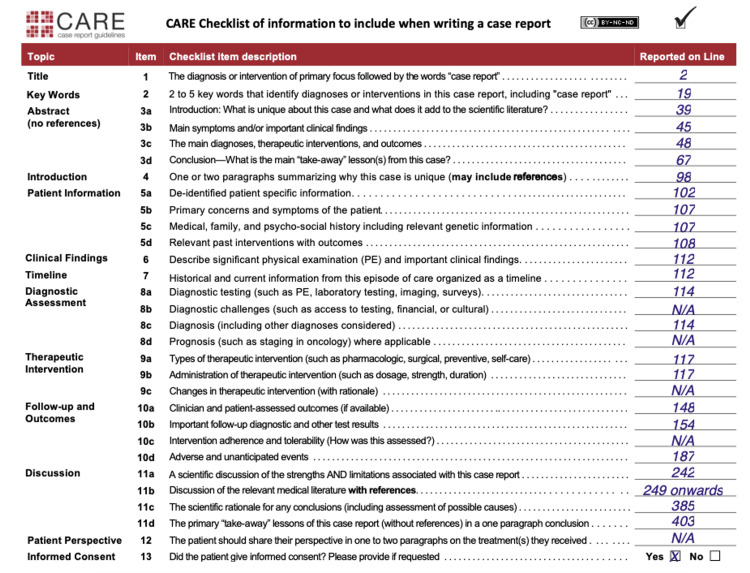
CARE checklist

## Appendices

**Table 10:** Complete search strategy

#	Searches	Results
1	(bilateral or simultaneous).mp. [mp=title, book title, abstract, original title, name of substance word, subject heading word, floating sub-heading word, keyword heading word, organism supplementary concept word, protocol supplementary concept word, rare disease supplementary concept word, unique identifier, synonyms, population supplementary concept word, anatomy supplementary concept word]	606134
2	(dislocat* or subluxation).mp. [mp=title, book title, abstract, original title, name of substance word, subject heading word, floating sub-heading word, keyword heading word, organism supplementary concept word, protocol supplementary concept word, rare disease supplementary concept word, unique identifier, synonyms, population supplementary concept word, anatomy supplementary concept word]	98157
3	(shoulder* or glenohumeral).mp. [mp=title, book title, abstract, original title, name of substance word, subject heading word, floating sub-heading word, keyword heading word, organism supplementary concept word, protocol supplementary concept word, rare disease supplementary concept word, unique identifier, synonyms, population supplementary concept word, anatomy supplementary concept word]	112135
4	((bilateral or simultaneous or both) adj2 (dislocat* or subluxation) adj2 (shoulder* or glenohumeral)).mp.	135
5	(unilateral or single).mp. [mp=title, book title, abstract, original title, name of substance word, subject heading word, floating sub-heading word, keyword heading word, organism supplementary concept word, protocol supplementary concept word, rare disease supplementary concept word, unique identifier, synonyms, population supplementary concept word, anatomy supplementary concept word]	2499732
6	4 and 5	13
7	limit 4 to last 10 years	53
8	1 and 2 and 3	707
9	(complicat* or manage* or "risk factor*" or outcome*).mp. [mp=title, book title, abstract, original title, name of substance word, subject heading word, floating sub-heading word, keyword heading word, organism supplementary concept word, protocol supplementary concept word, rare disease supplementary concept word, unique identifier, synonyms, population supplementary concept word, anatomy supplementary concept word]	8758169
10	8 and 9	456
11	limit 10 to last 10 years	218
12	("bilateral shoulder dislocation" or "bilateral dislocation of the shoulder" or "bilateral shoulder subluxation").mp. [mp=title, book title, abstract, original title, name of substance word, subject heading word, floating sub-heading word, keyword heading word, organism supplementary concept word, protocol supplementary concept word, rare disease supplementary concept word, unique identifier, synonyms, population supplementary concept word, anatomy supplementary concept word]	43
13	(trauma* or acute).mp. [mp=title, book title, abstract, original title, name of substance word, subject heading word, floating sub-heading word, keyword heading word, organism supplementary concept word, protocol supplementary concept word, rare disease supplementary concept word, unique identifier, synonyms, population supplementary concept word, anatomy supplementary concept word]	2175215
14	12 and 13	15

**Table 11:** Summary of reports of simultaneous bilateral shoulder dislocation

Case Report	Sex & Age	Dislocation	Aetiology	Associated Injury	Imaging	Management
[3]	19M	Anterior	Trauma	Prox hum #, GT #	XR	CR
[4]	61F	Anterior	Trauma	Nil	XR	CR
[8]	20M	Anterior	Trauma	Nil	XR, EMG	OR
[9]	52M	Anterior	Trauma	Hum head #	XR	ORIF
[10]	40M	Anterior	Trauma	Nil	XR, CT	ORIF
[11]	31M	Anterior	Trauma	GT #	XR	ORIF
[12]	28M	Anterior	Trauma	Coracoid #, Hill-Sachs #, Bicep rupture	XR, CT	ORIF
[13]	68M	Posterior	Trauma	Hum head #, Hill-Sachs #	XR, CT	ORIF
[14]	29M	Anterior	Trauma	Nil	XR	CR
[15]	21M	Anterior	Trauma	Nil	XR	CR
[16]	21M	Anterior	Trauma	Nil	XR	CR
[17]	35M	Antero-posterior	Trauma	GT #	XR	ORIF
[18]	29M	Posterior	Trauma	Prox hum #	XR, CT	ORIF
[19]	45M	Posterior	Trauma	Tuberculum minus #, Hill-Sachs #	XR, CT	CR
[20]	46M	Anterior	Trauma	Nil	XR	CR
[21]	30M	Anterior	Trauma	GT #	XR	CR
[22]	55M	Posterior	Trauma	Hum head #, Hill-Sachs #	XR, CT	ORIF
[23]	23M	Anterior	Trauma	Nil	XR	CR
[24]	33M	Anterior	Trauma	Hum head #	XR, CT, MRI	CR + ORIF
[25]	33M	Anterior	Trauma	GT #, Hum head #	XR, CT	CR
[26]	36M	Inferior	Trauma	GT #, Prox hum #	XR	CR + ORIF
[27]	39M	Anterior	Trauma	Nil	XR, CT	CR
[28]	67M	Anterior	Trauma	RTC, Hum head #, Hill-Sachs #	XR	CR + ORIF
[29]	72M	Anterior	Trauma	Hum head #	XR, CT	ORIF
[30]	35M	Anterior	Trauma	GT #	XR	CR + OR
[31]	36M	Antero-posterior	Trauma	GT #, Prox hum #	XR, CT	CR + ORIF
[32]	66F	Anterior	Trauma	Hum neck #	XR, CT	CR + ORIF
[33]	69F	Anterior	Trauma	GT #	XR, MRI	CR + ORIF
[34]	52F	Anterior	Trauma	Hum head #	Unavailable	ORIF
[35]	59F	Inferior	Trauma	RCT	XR, MRI	CR
[36]	41F	Anterior	Trauma	GT #, Nerve injury (brachial plexus)	XR, CT, EMG	CR
[37]	16F	Anterior	Trauma	Hill-Sachs #	XR	CR
[38]	84F	Anterior	Trauma	Nerve Injury (axillary & radial)	XR, EMG	CR
